# Primary central nervous system Burkitt lymphoma in a 38-year-old immunocompetent woman: A case report

**DOI:** 10.1097/MD.0000000000042321

**Published:** 2025-04-25

**Authors:** Kun Xue, Anling Zhang, Xu Yan, Shuyu Liu, Dawei Chen

**Affiliations:** a Department of Neurosurgery, The First Hospital of Jilin University, Changchun, Jilin, China; b Department of Stomatology, Jilin Province First Automobile Workshop General Hospital, Changchun, Jilin, China; c Department of Pathology, The First Hospital of Jilin University, Changchun, Jilin, China.

**Keywords:** Burkitt lymphoma, chemotherapy, magnetic resonance imaging, meningioma, primary central nervous system lymphomas

## Abstract

**Rationale:**

Primary central nervous system Burkitt lymphoma (PCNSBL) is a rare and aggressive malignancy, particularly challenging to diagnose in immunocompetent individuals due to its nonspecific presentation.

**Patient concerns:**

A 38-year-old immunocompetent woman presented with a 1-week history of progressively severe headaches in the left frontotemporal region, without systemic symptoms or significant laboratory abnormalities.

**Diagnoses:**

Advanced magnetic resonance imaging revealed a nodular lesion in the left frontal area, initially diagnosed as meningioma. Postsurgical histopathological analysis confirmed the diagnosis of Burkitt lymphoma, characterized by diffuse infiltration of medium-sized lymphocytes, a high MIB1 proliferation index, and Myc gene rearrangement.

**Interventions:**

The patient underwent complete surgical resection of the tumor and a 5-cycle chemotherapy regimen based on high-dose methotrexate, without the need for radiotherapy due to the localized nature of the tumor and complete surgical removal.

**Outcomes:**

Postoperatively, the patient’s headaches resolved, and no evidence of tumor recurrence was observed on magnetic resonance imaging after 11 months of follow-up. Additional examinations, including fluorodeoxyglucose-18–positron emission computed tomography, bone marrow biopsy, and cerebrospinal fluid cytology, confirmed the absence of systemic involvement.

**Lessons:**

This case highlights the importance of considering PCNSBL in the differential diagnosis of brain tumors, even in immunocompetent patients. Early diagnosis and a tailored chemotherapy regimen can lead to favorable treatment outcomes, emphasizing the need for a multimodality approach in managing PCNSBL.

## 1. Introduction

Primary central nervous system lymphoma (PCNSL), a type of rare brain cancer, comprises 6.7% of all primary tumors within the cranial cavity, with 95% classified as diffuse large B-cell lymphoma.^[1,2]^ Primary central nervous system Burkitt lymphoma (PCNSBL), an exceptionally rare subtype, represents a highly aggressive variant of non-Hodgkin lymphoma that typically originates from B cells in the germinal center. PCNSBL is primarily diagnosed in pediatric and adolescent populations, and it presents diverse clinical manifestations and poses diagnostic challenges.^[3]^ The incidence of PCNSBL and its association with Epstein–Barr virus (EBV) vary among different populations. For instance, in Central Africa, endemic Burkitt lymphoma is highly associated with EBV infection, whereas in Western Europe and the United States, the sporadic form of Burkitt lymphoma, which is less frequently linked to EBV infection, prevails. Additionally, Burkitt lymphoma occurs among immunocompromised patients such as those with HIV infection or organ transplant recipients. Although approximately 90% of cases of Burkitt lymphoma feature C-myc gene rearrangement, the exact pathogenic mechanisms remain largely obscure.^[4]^ This case report detailed a rare case of dural PCNSBL and, through a review of the literature, discussed its clinical characteristics, diagnostic challenges, and therapeutic strategies.

## 2. Case presentation

### 2.1. History

A 38-year-old woman presented with a 1-week history of progressively worsening blunt pain in the left frontotemporal region accompanied by radiating pain to the eye without any apparent trigger. She did not manifest any signs of fever, nausea, or weight reduction. Her medical background was unexceptional and devoid of any immune system disorders, transplants, or autoimmune diseases.

### 2.2. Examination findings

**Figure 1.**
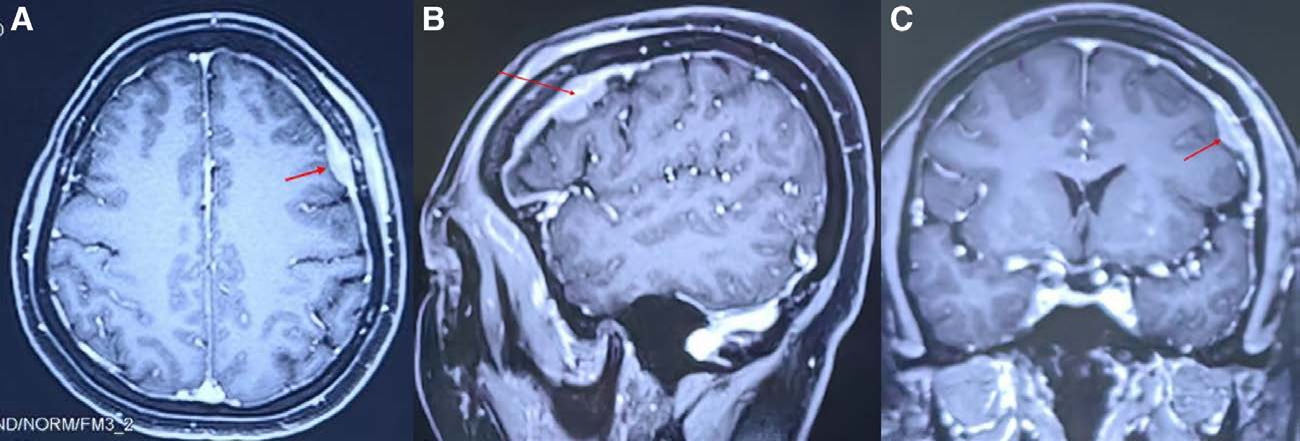
(A–C) MRI of the head revealed a nodular lesion located below the skull plate in the left frontal area. The lesion displayed moderate uniform enhancement upon contrast scans, and the adjacent dura mater exhibited thickening and enhancement.

Physical examination revealed distress but no generalized lymphadenopathy. She was conscious with fluent speech, and a neurological examination did not disclose any abnormalities. The results of routine laboratory tests such as a full blood count, evaluations of liver and renal function, and measurements of lactate dehydrogenase were all within the expected ranges. Tests for HIV, HBV, HCV antibodies, and EBV were all negative. Immunoglobulin and complement levels were normal. Computed tomography (CT) of the cervical, thoracic, abdominal, and pelvic regions demonstrated a lack of any abnormal findings. Brain magnetic resonance imaging (MRI) revealed a 1.8 × 1.0 × 0.9 cm^3^ space-occupying lesion below the skull plate in the left frontal area with moderate, uniform enhancement on contrast and adjacent thickening and enhancement of the dura mater (Fig. 1A–C).

### 2.3. Treatment course

**Figure 2.**
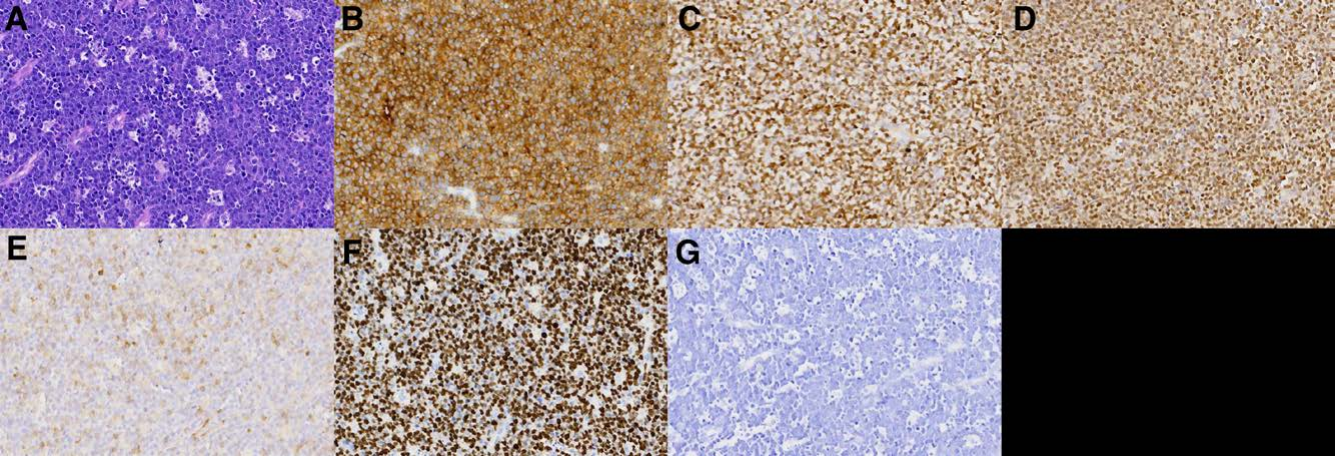
Histology and immunohistochemistry. (A) Diffuse infiltration of medium-sized lymphocytes, displaying a “starry sky” pattern (H&E staining, ×200 magnification). (B) Positive CD20 expression on tumor cell membranes (×400 magnification). (C) Bcl-6 positivity. (D) Confirmation of Myc gene rearrangement. (E) Bcl-2 positivity. (F) More than 95% of cells exhibited Ki-67 expression (magnification × 400). (G) EBER test negativity. EBER = Epstein–Barr encoding region.

The preliminary diagnosis was left frontal meningioma. Surgery was performed to fully excise the tumor and the affected dura mater. Intraoperatively, the tumor’s base was located on the dura mater, which was grayish-white, firm, and moderately vascular. There was no evidence of local skull involvement. The lesion, encompassing the adjacent thickened dura mater, was extensively resected without the utilization of rapid frozen pathological assessment. The histopathological analysis postsurgery disclosed widespread invasion by medium-sized lymphocytes, creating the distinctive “starry sky” appearance (Fig. 2A). Immunohistochemistry was positive for CD10, CD20, CD79a, and Bcl-6 and negative for Bcl-2, CD2, CD99, CD117, and MUM1. The MIB1 proliferation index was up to 99%. The Epstein–Barr encoding region test was negative, and fluorescence in situ hybridization confirmed Myc gene rearrangement (Fig. 2B–G), establishing a diagnosis of Burkitt lymphoma. Then, the patient underwent chemotherapy based on the G-CODOX regimen for 5 cycles every 21 days, during which no grade 2 or higher toxic reactions were observed. Given the tumor’s limited spread and comprehensive surgical removal, as well as to avoid potential neurocognitive and psychological disorders from radiation therapy, the patient did not receive radiotherapy.

### 2.4. Follow-up

**Figure 3.**
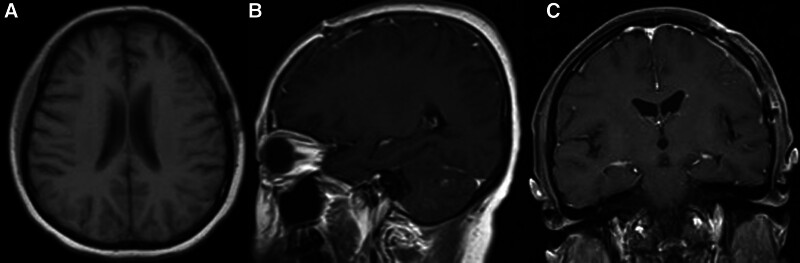
(A–C) Eleven months after surgery, a follow-up MRI revealed no signs of tumor recurrence.

The patient’s headaches resolved without postoperative complications. Further ophthalmic examination (slit-lamp examination), fluorodeoxyglucose-18 (FDG)–positron emission tomography (PET), bone marrow biopsy, and cerebrospinal fluid cytology revealed no abnormalities, confirming the diagnosis of dural PCNSBL. Eleven months postsurgery, MRI indicated no signs of tumor recurrence (Fig. 3A–C).

## 3. Discussion

PCNSL represents 7% of all newly diagnosed central nervous system tumors, and PCNSBL comprises only 3% to 5% of PCNSL cases.^[5]^ Worldwide, <40 cases of intracranial and intraspinal PCNSBL have been documented in the literature, including this case. Predominantly, these tumors arise within the brain parenchyma, although rarer locations such as the optic nerve, pituitary gland, cavernous sinus, brain ventricles, dura mater, and extracranial epidural space have also been reported.^[5–12]^ The condition affects individuals across all age groups, predominantly adults, with a slight male predominance (2:1). Clinical manifestations range from symptoms suggestive of elevated intracranial pressure, such as headaches, nausea, and vomiting, to localized neurological impairments like weakness and speech disorders. PCNSBL is an exceptionally rare condition. Menniti and colleagues^[13]^ detailed a case of primary dural mucosa-associated lymphoid tissue lymphoma in the left frontoparietal region, which mimicked convexity meningioma and responded well to surgical excision. Gobbato et al^[14]^ reported a patient with HIV who presented with PCNSBL of the right frontotemporoparietal dura; unfortunately, the patient died from acute respiratory failure following surgery. Li et al^[15]^ described a large PCNSL adjacent to the falx cerebri in the frontal lobe, which was initially indistinguishable from meningioma, and the patient exhibited a positive response to complete surgical resection and chemotherapy. Jouda et al^[16]^ documented a case of PCNSL in the left temporal lobe that resembled meningioma, and the patient benefited from a multimodality approach involving surgery and chemoradiotherapy, leading to a favorable therapeutic outcome. However, this case exhibited atypical clinical features with no systemic symptoms and negative laboratory findings, complicating the diagnosis. Radiological investigations revealed a solitary meningeal lesion without the multicentric infiltration typical of systemic Burkitt lymphoma. Further examinations, including slit-lamp examination, FDG–PET, bone marrow biopsy, and cerebrospinal fluid cytology, excluded central nervous system involvement in systemic Burkitt lymphoma. Ultimately, postoperative histopathology confirmed the diagnosis of Burkitt lymphoma, underscoring the necessity for a broad differential diagnosis and thorough investigations when facing atypical clinical presentations.

On MRI, PCNSBL appears nonspecific, and it can be easily confused with other brain tumors. Lesions typically manifest in cerebral hemispheres, corpus callosum, and periventricular white matter, displaying isointense to hypointense signals on T1-weighted imaging and isointense to mildly hypointense signals on T2-weighted imaging, as well as homogeneous nodular or mass-like enhancement following contrast administration.^[17]^ Magnetic resonance spectroscopy observations of an increased choline peak, slightly decreased N-acetylaspartate peak, and significantly elevated lipid peak, aiding in diagnosis.^[18]^ FDG uptake on PET–CT is crucial for lymphoma staging and treatment assessment. The differential diagnosis should exclude meningioma, meningeal marginal zone lymphoma, and hemangiopericytoma, due to distinct MRI signal characteristics and enhancement patterns.^[19]^ For instance, meningiomas often present as broad-based nodules, possibly with calcifications and the “dural tail sign,” whereas meningeal marginal zone lymphoma exhibits flat lesions that enhance significantly post-contrast, possibly involving adjacent bone and brain parenchyma. Hemangiopericytomas appear as narrow-based nodules with mixed signal intensity on MRI and marked enhancement post-contrast administration. Given the overlapping imaging features between PCNSBL with other conditions, accurate diagnosis necessitates integrating multiple imaging modalities alongside clinical context. On MRI, the lesion had hypointense to isointense signals on T1-weighted imaging and isointense to slightly hyperintense signals on T2-weighted imaging. The brain parenchyma was mildly compressed without significant cerebral edema, and there was no evidence of local bone involvement. Following contrast administration, the tumor and surrounding thickened dura mater displayed moderate enhancement, but the “dural tail” sign was conspicuously absent. Owing to the limited awareness of this condition, advanced diagnostic studies such as magnetic resonance spectroscopy and PET–CT were not undertaken, leading to an initial assessment that heavily leaned toward a meningioma diagnosis. The postsurgical histopathology confirmed Burkitt lymphoma, highlighting the importance of maintaining broad differential diagnostic considerations in the face of atypical imaging findings.

Histopathological examination is crucial for the diagnosis of PCNSBL, which is characterized by the diffused infiltration of medium-sized round lymphocytes, creating a distinctive “starry sky” appearance, along with high mitotic activity and frequently observed mitotic figures.^[20]^ The MIB1 proliferation index is typically high (99%–100%), reflecting the tumor’s high aggressiveness and rapid growth characteristics. Immunohistochemistry aids in diagnosis, including positive expression of CD19, CD20, CD22, CD10, and CD79a, whereas CD5, CD23, Bcl-2, and TdT are usually negative, assisting in differentiating PCNSBL from other lymphomas and leukemia subtypes.^[21]^ Molecular genetic testing, particularly the rearrangement status of the Myc gene, provides critical diagnostic information for PCNSBL. Myc gene rearrangement, a characteristic molecular genetic alteration in Burkitt lymphoma, was confirmed in this case by fluorescence in situ hybridization, underscoring its importance in the pathological diagnosis. Understanding the histological and molecular genetic features of PCNSBL is crucial for clinical decision-making, guiding treatment selection such as targeted therapy strategies for Myc gene rearrangement and facilitating prognosis assessment and patient management.

Chemotherapy, particularly regimens featuring high-dose methotrexate (HD-MTX), augmented by rituximab, is central to treating PCNSBL due to its documented enhancements in progression-free survival (PFS), overall response rates, and overall survival.^[22–24]^ Protocols vary, mirroring those used for systemic Burkitt lymphoma and primary CNS lymphoma, and include HD-MTX with or without cyclophosphamide, doxorubicin, vincristine, and prednisone. Treatment may also involve surgical interventions, radiotherapy, whole-body/intrathecal chemotherapy, and the Ferreri regimen.^[25–27]^ Adjustments are tailored to patient factors, tumor characteristics, and response. In this instance, the G-CODOX protocol, which omits radiotherapy, was chosen due to the localized nature of the tumor and comprehensive surgical resection. MRI imaging and symptom improvement evaluation confirmed treatment efficacy for the patient’s postoperative headache symptoms resolved without complications after 11 months of rigorous monitoring, affirming the treatment efficacy.

Surgical intervention assumes a pivotal role in PCNSBL management by enabling lymphoma diagnosis and alleviating intracranial pressure-related symptoms through histological evidence and symptom relief.^[28]^ Surgical suitability hinges on factors like age, health status, tumor characteristics, and location. In cases of isolated superficial PCNSBL presenting with increased intracranial pressure, focal neurological deficits, or spinal cord compression, surgical tumor resection is instrumental in swiftly resolving the mass effect, alleviating symptoms, and facilitating neurological recovery. Complete tumor resection has yielded better PFS and overall survival than subtotal resection or biopsy. Furthermore, the administration of postoperative chemotherapy in conjunction with surgery has been demonstrated to significantly prolong PFS compared with chemotherapy alone.^[29–31]^ Despite advancements ensuring surgical safety, careful risk-benefit assessment is crucial due to potential complications such as infection, hemorrhage, and neural damage leading to postoperative neurologic deficits. Comprehensive management strategies are facilitated by surgical outcomes guiding subsequent chemotherapeutic and radiotherapeutic decisions, necessitating integrated adjuvant chemotherapy and potential radiotherapy for residual tumor cells and relapse prevention.^[32,33]^

This case report provided detailed clinical observations and treatment experiences for dural PCNSBL. However, its single-case design limits generalizability across the patient community. The lack of a control group posed challenges in accurately assessing treatment effectiveness and safety. Additionally, the constrained follow-up duration impeded comprehensive long-term efficacy and survivorship assessments, necessitating prolonged follow-up in future research. Future studies should prioritize multicentric, prospective investigations to validate these findings and establish long-term treatment efficacy. Exploring novel treatment avenues, such as targeted and immunotherapies, could broaden therapeutic options for patients with PCNSBL. Creating precise prognostic models is crucial for predicting treatment outcomes and optimizing personalized treatment strategies. Furthermore, enhancing international collaboration and data sharing will deepen our understanding of the clinical characteristics and treatment responses of PCNSBL, propelling the development of new therapies and optimizing existing treatments.

This case report underscored the significance of prompt and precise diagnostic procedures and demonstrated the effectiveness of surgical intervention combined with HD-MTX-based chemotherapy in managing PCNSBL. However, further research and extended follow-up are imperative for standardizing treatment protocols and prognostic assessments.

## Acknowledgments

This work was supported by the Jilin Provincial Department of Science and Technology’s special fund for the development of the medical and health industry (Grant No. 20210401137YY).

## Author contributions

**Formal analysis:** Anling Zhang, Xu Yan.

**Visualization:** Anling Zhang.

**Writing – original draft:** Kun Xue, Shuyu Liu.

**Writing – review & editing:** Anling Zhang, Xu Yan, Dawei Chen.
